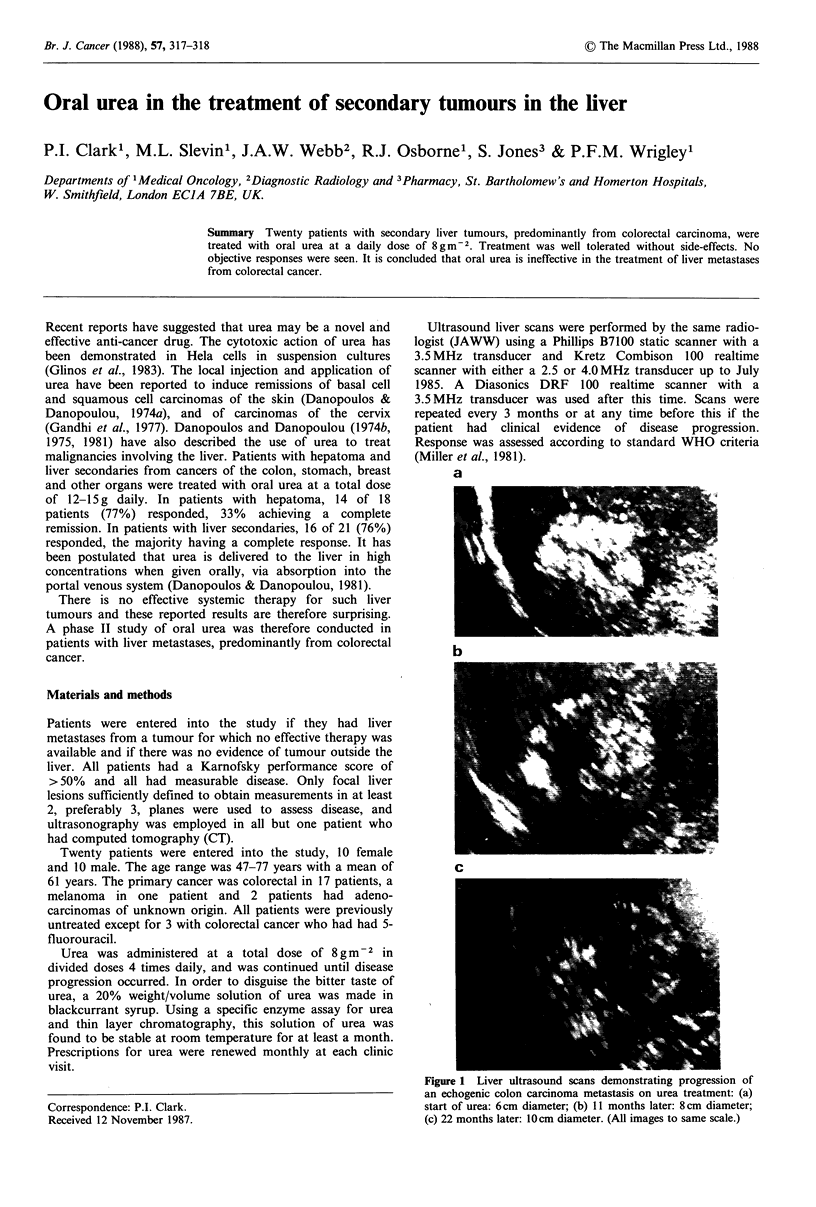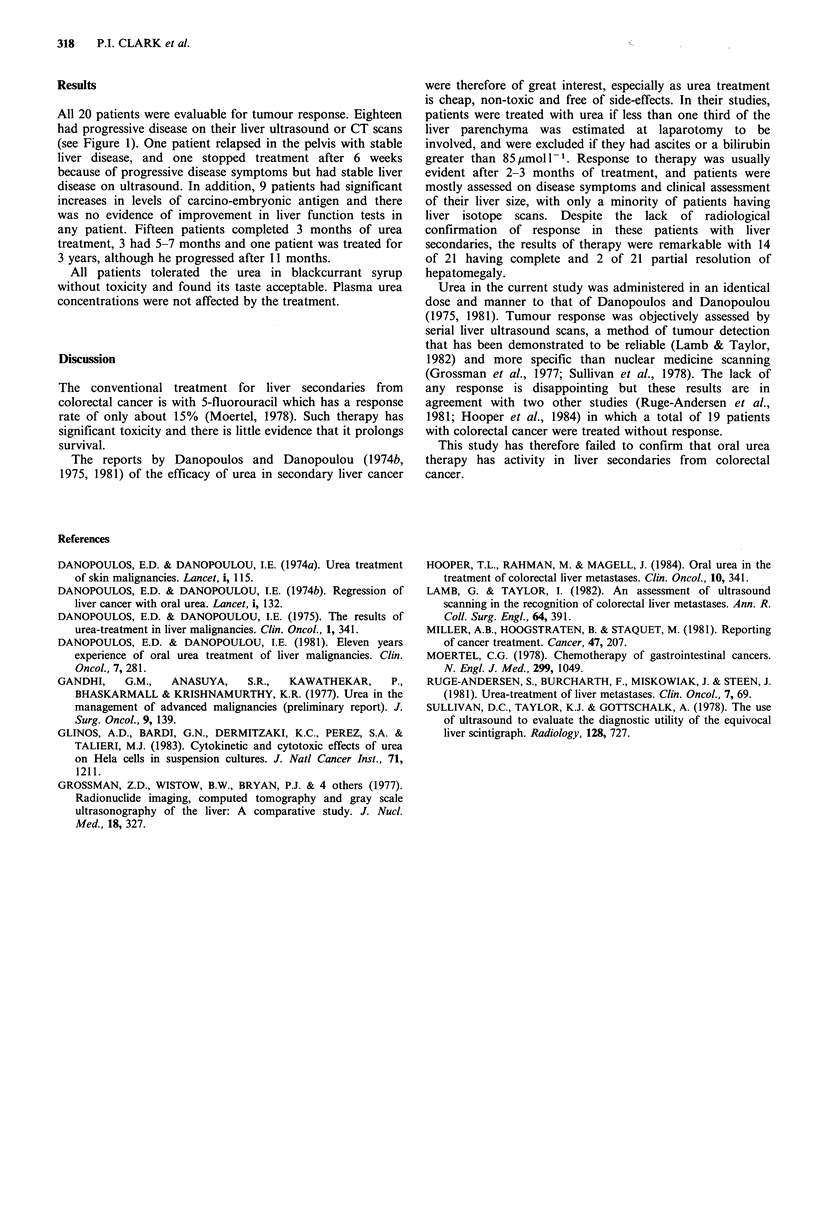# Oral urea in the treatment of secondary tumours in the liver.

**DOI:** 10.1038/bjc.1988.70

**Published:** 1988-03

**Authors:** P. I. Clark, M. L. Slevin, J. A. Webb, R. J. Osborne, S. Jones, P. F. Wrigley

**Affiliations:** Department of Medical Oncology, St. Bartholomew's Hospital, London, UK.

## Abstract

**Images:**


*Br. J. Cancer* (1988), **57**, 317–318 © The Macmillan Press Ltd., 1988

### Oral urea in the treatment of secondary tumours in the liver

P.I. Clark[1], M.L. Slevin[1], J.A.W. Webb[2], R.J. Osborne[1], S. Jones[3] & P.F.M. Wrigley[1]

*Departments of [1]Medical Oncology, [2]Diagnostic Radiology and [3]Pharmacy, St. Bartholomew's and Homerton Hospitals, W. Smithfield, London EC1A 7BE, UK.*

**Summary** Twenty patients with secondary liver tumours, predominantly from colorectal carcinoma, were treated with oral urea at a daily dose of $8\,gm^{-2}$. Treatment was well tolerated without side-effects. No objective responses were seen. It is concluded that oral urea is ineffective in the treatment of liver metastases from colorectal cancer.

Recent reports have suggested that urea may be a novel and effective anti-cancer drug. The cytotoxic action of urea has been demonstrated in Hela cells in suspension cultures (Glinos *et al.*, 1983). The local injection and application of urea have been reported to induce remissions of basal cell and squamous cell carcinomas of the skin (Danopoulos & Danopoulou, 1974*a*), and of carcinomas of the cervix (Gandhi *et al.*, 1977). Danopoulos and Danopoulou (1974*b*, 1975, 1981) have also described the use of urea to treat malignancies involving the liver. Patients with hepatoma and liver secondaries from cancers of the colon, stomach, breast and other organs were treated with oral urea at a total dose of 12–15 g daily. In patients with hepatoma, 14 of 18 patients (77%) responded, 33% achieving a complete remission. In patients with liver secondaries, 16 of 21 (76%) responded, the majority having a complete response. It has been postulated that urea is delivered to the liver in high concentrations when given orally, via absorption into the portal venous system (Danopoulos & Danopoulou, 1981).

There is no effective systemic therapy for such liver tumours and these reported results are therefore surprising. A phase II study of oral urea was therefore conducted in patients with liver metastases, predominantly from colorectal cancer.

### Materials and methods

Patients were entered into the study if they had liver metastases from a tumour for which no effective therapy was available and if there was no evidence of tumour outside the liver. All patients had a Karnofsky performance score of >50% and all had measurable disease. Only focal liver lesions sufficiently defined to obtain measurements in at least 2, preferably 3, planes were used to assess disease, and ultrasonography was employed in all but one patient who had computed tomography (CT).

Twenty patients were entered into the study, 10 female and 10 male. The age range was 47–77 years with a mean of 61 years. The primary cancer was colorectal in 17 patients, a melanoma in one patient and 2 patients had adenocarcinomas of unknown origin. All patients were previously untreated except for 3 with colorectal cancer who had had 5-fluorouracil.

Urea was administered at a total dose of $8\,gm^{-2}$ in divided doses 4 times daily, and was continued until disease progression occurred. In order to disguise the bitter taste of urea, a 20% weight/volume solution of urea was made in blackcurrant syrup. Using a specific enzyme assay for urea and thin layer chromatography, this solution of urea was found to be stable at room temperature for at least a month. Prescriptions for urea were renewed monthly at each clinic visit.

Ultrasound liver scans were performed by the same radiologist (JAWW) using a Phillips B7100 static scanner with a 3.5 MHz transducer and Kretz Combison 100 realtime scanner with either a 2.5 or 4.0 MHz transducer up to July 1985. A Diasonics DRF 100 realtime scanner with a 3.5 MHz transducer was used after this time. Scans were repeated every 3 months or at any time before this if the patient had clinical evidence of disease progression. Response was assessed according to standard WHO criteria (Miller *et al.*, 1981).

**Figure 1** Liver ultrasound scans demonstrating progression of an echogenic colon carcinoma metastasis on urea treatment: (a) start of urea: 6 cm diameter; (b) 11 months later: 8 cm diameter; (c) 22 months later: 10 cm diameter. (All images to same scale.)

Correspondence: P.I. Clark.
Received 12 November 1987.

### Results

All 20 patients were evaluable for tumour response. Eighteen had progressive disease on their liver ultrasound or CT scans (see Figure 1). One patient relapsed in the pelvis with stable liver disease, and one stopped treatment after 6 weeks because of progressive disease symptoms but had stable liver disease on ultrasound. In addition, 9 patients had significant increases in levels of carcino-embryonic antigen and there was no evidence of improvement in liver function tests in any patient. Fifteen patients completed 3 months of urea treatment, 3 had 5–7 months and one patient was treated for 3 years, although he progressed after 11 months.

All patients tolerated the urea in blackcurrant syrup without toxicity and found its taste acceptable. Plasma urea concentrations were not affected by the treatment.

### Discussion

The conventional treatment for liver secondaries from colorectal cancer is with 5-fluorouracil which has a response rate of only about 15% (Moertel, 1978). Such therapy has significant toxicity and there is little evidence that it prolongs survival.

The reports by Danopoulos and Danopoulou (1974*b*, 1975, 1981) of the efficacy of urea in secondary liver cancer were therefore of great interest, especially as urea treatment is cheap, non-toxic and free of side-effects. In their studies, patients were treated with urea if less than one third of the liver parenchyma was estimated at laparotomy to be involved, and were excluded if they had ascites or a bilirubin greater than $85\,\mu mol\,l^{-1}$. Response to therapy was usually evident after 2–3 months of treatment, and patients were mostly assessed on disease symptoms and clinical assessment of their liver size, with only a minority of patients having liver isotope scans. Despite the lack of radiological confirmation of response in these patients with liver secondaries, the results of therapy were remarkable with 14 of 21 having complete and 2 of 21 partial resolution of hepatomegaly.

Urea in the current study was administered in an identical dose and manner to that of Danopoulos and Danopoulou (1975, 1981). Tumour response was objectively assessed by serial liver ultrasound scans, a method of tumour detection that has been demonstrated to be reliable (Lamb & Taylor, 1982) and more specific than nuclear medicine scanning (Grossman *et al.*, 1977; Sullivan *et al.*, 1978). The lack of any response is disappointing but these results are in agreement with two other studies (Ruge-Andersen *et al.*, 1981; Hooper *et al.*, 1984) in which a total of 19 patients with colorectal cancer were treated without response.

This study has therefore failed to confirm that oral urea therapy has activity in liver secondaries from colorectal cancer.

### References

DANOPOULOS, E.D. & DANOPOULOU, I.E. (1974*a*). Urea treatment of skin malignancies. *Lancet*, **i**, 115.

DANOPOULOS, E.D. & DANOPOULOU, I.E. (1974*b*). Regression of liver cancer with oral urea. *Lancet*, **i**, 132.

DANOPOULOS, E.D. & DANOPOULOU, I.E. (1975). The results of urea-treatment in liver malignancies. *Clin. Oncol.*, **1**, 341.

DANOPOULOS, E.D. & DANOPOULOU, I.E. (1981). Eleven years experience of oral urea treatment of liver malignancies. *Clin. Oncol.*, **7**, 281.

GANDHI, G.M., ANASUYA, S.R., KAWATHEKAR, P., BHASKARMALL & KRISHNAMURTHY, K.R. (1977). Urea in the management of advanced malignancies (preliminary report). *J. Surg. Oncol.*, **9**, 139.

GLINOS, A.D., BARDI, G.N., DERMITZAKI, K.C., PEREZ, S.A. & TALIERI, M.J. (1983). Cytokinetic and cytotoxic effects of urea on Hela cells in suspension cultures. *J. Natl Cancer Inst.*, **71**, 1211.

GROSSMAN, Z.D., WISTOW, B.W., BRYAN, P.J. & 4 others (1977). Radionuclide imaging, computed tomography and gray scale ultrasonography of the liver: A comparative study. *J. Nucl. Med.*, **18**, 327.

HOOPER, T.L., RAHMAN, M. & MAGELL, J. (1984). Oral urea in the treatment of colorectal liver metastases. *Clin. Oncol.*, **10**, 341.

LAMB, G. & TAYLOR, I. (1982). An assessment of ultrasound scanning in the recognition of colorectal liver metastases. *Ann. R. Coll. Surg. Engl.*, **64**, 391.

MILLER, A.B., HOOGSTRATEN, B. & STAQUET, M. (1981). Reporting of cancer treatment. *Cancer*, **47**, 207.

MOERTEL, C.G. (1978). Chemotherapy of gastrointestinal cancers. *N. Engl. J. Med.*, **299**, 1049.

RUGE-ANDERSEN, S., BURCHARTH, F., MISKOWIAK, J. & STEEN, J. (1981). Urea-treatment of liver metastases. *Clin. Oncol.*, **7**, 69.

SULLIVAN, D.C., TAYLOR, K.J. & GOTTSCHALK, A. (1978). The use of ultrasound to evaluate the diagnostic utility of the equivocal liver scintigraph. *Radiology*, **128**, 727.